# Management of RAASi-associated hyperkalemia in patients with cardiovascular disease

**DOI:** 10.1007/s10741-020-10069-3

**Published:** 2021-02-18

**Authors:** José Silva-Cardoso, Dulce Brito, João Miguel Frazão, Aníbal Ferreira, Paulo Bettencourt, Patrícia Branco, Cândida Fonseca

**Affiliations:** 1 Heart Failure and Transplant Clinic, Cardiology Service, São João University Hospital Centre, Porto, Portugal; 2 Faculty of Medicine, University of Porto, Porto, Portugal; 3 CINTESIS – Centre for Health Technology and Services Research, Porto, Portugal; 4 Serviço de Cardiologia, Centro Hospitalar Universitário de Lisboa Norte, Lisbon, Portugal; 5 CCUL, Faculdade de Medicina, Universidade de Lisboa, Lisbon, Portugal; 6 Instituto de Investigação e Inovação em Saúde e Instituto de Engenharia Biomédica (INEB), Universidade do Porto, Porto, Portugal; 7 Serviço de Nefrologia, Centro Hospitalar Universitário São João, Faculdade de Medicina, Universidade Do Porto, Porto , Portugal; 8 Departamento de Nefrologia, Centro Hospitalar e Universitário de Lisboa Central, Lisbon, Portugal; 9 NOVA Medical School, Faculdade de Ciências Médicas, Universidade Nova de Lisboa, Lisbon, Portugal; 10 Cardiovascular Research Centre, Universidade do Porto, Porto, Portugal; 11 Departamento de Nefrologia, Hospital de Santa Cruz, CHLO, Lisbon, Portugal; 12 Heart Failure Clínic, Hospital São Francisco Xavier, Centro Hospitalar de Lisboa Ocidental, Lisbon, Portugal

**Keywords:** Novel potassium binders, Heart failure with reduced ejection fraction, Renin–angiotensin–aldosterone system inhibitors, Hyperkalemia, RAASi optimization

## Abstract

Renin–angiotensin–aldosterone system inhibitors (RAASi) reduce morbidity and mortality in heart failure (HF) with reduced ejection fraction in a dose-dependent manner. They also have a positive impact in other cardiovascular diseases (CVDs). However, RAASi may induce hyperkalemia, a potentially life-threatening disorder. This risk is further increased in those with concomitant chronic kidney disease, diabetes mellitus, and/or in patients with hypertension. Current treatment guidelines recommend maximal RAASi dosing to improve clinical outcomes; however, this is often limited by the development of hyperkalemia. When this occurs, current guidelines recommend RAASi down-titration/interruption, which, while improving short-term prognosis, is associated with a negative long-term prognostic impact. At present, the European Society of Cardiology suggests the consideration of novel potassium binders (patiromer and sodium zirconium cyclosilicate) for the management of RAASi-associated hyperkalemia. Both drugs can reduce serum potassium levels and prevent recurrent hyperkalemia. Additionally, patiromer showed enabling of RAASi optimization in high-risk patients. Nevertheless, precise recommendations on the use of these drugs are lacking. Building upon current HF guideline recommendations, a multidisciplinary expert panel convened to design an algorithm providing practical guidance on the use of novel potassium binders/patiromer in patients with HF and/or other CVD. As a result of that effort, we present an evidence-based treatment algorithm for the management of hyperkalemia with novel potassium binders/patiromer in patients with HF and/or other CVD receiving RAASi, including the necessary monitoring to avoid induction of hypokalemia. This algorithm aims to maintain or up-titrate RAASi to optimized doses, while maintaining normokalemia, improved clinical outcomes, and long-term prognosis.

## Introduction

Renin–angiotensin–aldosterone system inhibitors (RAASi) include angiotensin-converting enzyme inhibitors, angiotensin II receptor blockers, mineralocorticoid receptor antagonists, and angiotensin receptor-neprilysin inhibitors. RAASi are disease-modifying drugs in heart failure with reduced ejection fraction and have a positive impact in other cardiovascular conditions [1–3]. However, patients with cardiovascular disease (CVD) receiving treatment with RAASi are at increased risk of hyperkalemia, a potentially life-threatening disorder associated with cardiac arrhythmias and sudden cardiac death [2].

Hyperkalemia is usually classified as mild (serum potassium [K^+^] 5.1–5.5 mEq/L), moderate (serum K^+^ 5.6–6.0 mEq/L), or severe (serum K^+^  > 6.0 mEq/L) [1]. In the RALES study, only serum K^+^ levels above 5.5 mEq/L were associated with an increased risk of mortality [4]. While normokalemia is usually described as a serum K^+^ value of 3.5–5 mEq/L, in patients with heart failure (HF), chronic kidney disease (CKD), diabetes mellitus (DM), or a combination of these, recent data conclusively demonstrated a U-shaped curve of increased mortality associated with serum K^+^ values above or below 4.0–5.0 mEq/L [5]. Thus, while hyperkalemia is of concern, over-correction of this condition (serum K^+^  < 4.0 mEq/L) should be avoided at all costs in this patient group.

Incidence of hyperkalemia in patients with CVD receiving RAASi in real-world clinical practice has been shown to exceed that observed in clinical trials [6]. The risk of RAASi-induced hyperkalemia is particularly high in patients with HF and concomitant CKD and/or DM [3, 5]. Approximately 50% of these patients experience two or more recurrences of hyperkalemia within 1 year [2]. In this high-risk patient population, even mild hyperkalemia has been independently associated with a significantly increased risk of mortality [5]. Thus, while RAASi represent life-saving therapy, their use also poses a therapeutic dilemma—patients who benefit most from these treatments are typically those at the greatest risk of associated, severe hyperkalemic events [7].

Maximal RAASi dosing in patients with HF is advocated in treatment guidelines [1] and has been shown to improve survival [8]. However, following an episode of moderate or severe hyperkalemia, guidelines recommend the down-titration or cessation of RAASi, respectively [1, 2]. Once RAASi have been withheld, they are seldom re-initiated [9]. Real-world evidence has demonstrated that, compared with maximal target doses, sub-maximal RAASi dosing or RAASi discontinuation in patients with HF are associated with worse cardio-renal outcomes and an increased risk of hospitalization and mortality [8, 10]. In summary, while RAASi therapy improves long-term prognosis in a dose-dependent manner, increased serum K^+^ worsens short-term prognosis. The latter leads to RAASi dose reduction or withdrawal, which in turn is associated with worsened long-term prognosis.

Traditional options for the management of acute hyperkalemia are unsuitable for long-term use. However, two novel potassium-binding agents, patiromer and sodium zirconium cyclosilicate (SZC), have been approved recently, on the basis that they have been shown to normalize elevated serum K^+^ levels, maintain normokalemia over time, and prevent recurrent hyperkalemia [11–14].

SZC binds potassium through exchange with sodium, primarily in the gastrointestinal tract. In the phase III HARMONIZE trial, SZC demonstrated rapid normalization of serum potassium in patients with hyperkalemia and underlying CKD and/or DM and/or congestive HF, the majority of whom (68.8%) were on RAASi [11]. However, as the mechanism of action of SZC involves sodium, edema was reported in 2.4% of patients in the placebo group compared with up to 14.3% of patients who received the highest dose of SZC [11], which may constitute a limitation in hemodynamically unstable patients with HF presenting with volume overload. At present, there are no clinical trial data available for SZC and RAASi optimization in patients with HF and other comorbidities.

In contrast to SZC, the mechanism of action of patiromer does not involve sodium. In fact, patiromer binds potassium in exchange for calcium, mostly in the colon [12, 13]. Its sodium-free nature allows use in patients unable to tolerate an increase in sodium load, including those with HF and volume overload, hypertension, marked edema, or CKD. Clinical trials of patiromer in patients with CKD and HF have demonstrated rapid serum K^+^ normalization, reduced recurrent hyperkalemia, and RAASi enablement [13]. In the AMETHYST-DN trial, patiromer was shown to be effective in maintaining normal serum K^+^ levels for a period of 1 year in patients with DM and CKD receiving RAASi, with low incidence of hypokalemia [12]. The PEARL-HF study demonstrated the efficacy and safety of patiromer in preventing hyperkalemia in patients with HF, with or without kidney disease, who were started on spironolactone [13]. Hypomagnesia occurred in 24% of patients who received patiromer compared with 2% of patients who received the placebo (*p* = 0.001) [13], which highlights the need for serum magnesium monitoring in patients who receive this drug. Patiromer was also shown to enable continuation of treatment with spironolactone in the AMBER trial of patients with CKD and resistant hypertension [14]. The ongoing phase III DIAMOND study will evaluate the potential of patiromer to improve clinical outcomes in patients with HF by enabling long-term use of RAASi (ClinicalTrials.gov identifier: NCT03888066).

A recent consensus statement published by the Heart Failure Association (HFA) of the European Society of Cardiology (ESC) suggests that these novel potassium binders (patiromer and SZC) may be considered in patients with HF to manage hyperkalemia and, in selected circumstances, to enable the use of RAASi in more patients and at higher doses [15]. However, detailed recommendations for the use of these agents are not provided.

In the absence of clear guidance on the usage of these drugs, a multidisciplinary panel of expert physicians convened to develop an evidence-based algorithm to guide the prescription of the novel potassium binders/patiromer in everyday clinical practice. The designation “novel potassium binders/patiromer” was chosen due to the fact that at present, amongst novel potassium binders, only patiromer has published data on RAASi optimization in patients with HF and other comorbidities. The proposed treatment algorithm presented herein reflects the consensus opinions of all authors.

## Guidance on the management of chronic hyperkalemia in patients with CVD receiving RAASi

The expert panel proposes a treatment algorithm for chronic hyperkalemia that builds upon the 2016 ESC HF guidelines [1], the ESC Working Group on Cardiovascular Pharmacotherapy consensus on the management of hyperkalemia in patients with CVD treated with RAASi [2], and the HFA of the ESC clinical practice update on HF 2019 [15]. In line with these recommendations, the present algorithm aims to provide more detailed guidance with regard to the management of hyperkalemia, across the spectrum of severity, in patients with CVD treated with RAASi.

The guiding principle of the algorithm is to maintain or up-titrate RAASi therapy to maximum target doses in view of the prognostic gains achieved. In alignment with the aforementioned guidelines [1, 2, 15], we propose RAASi enablement and optimization in patients with HF and/or other CVD and chronic or recurrent hyperkalemia, through the use of approved, novel potassium-binding agents.

**Fig. 1:**
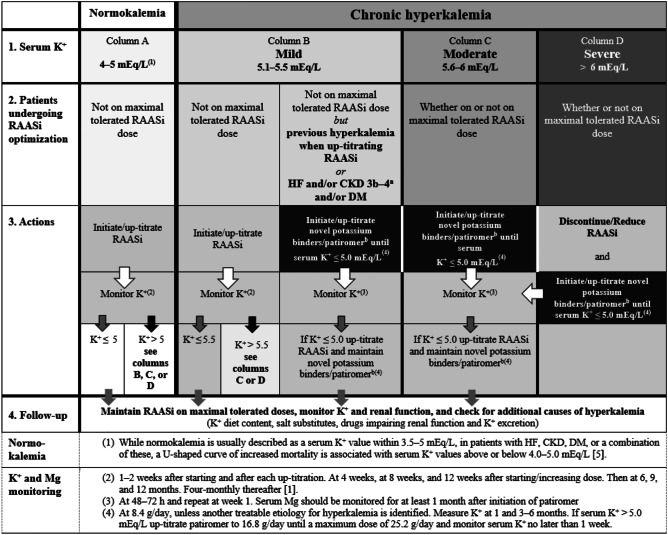
Treatment algorithm for the management of RAASi-associated hyperkalemia in patients with heart failure and/or other cardiovascular diseases ^a^CKD 3b–4 = eGFR > 15–45 mL/min/1.73 m^2^. ^b^Patiromer should be administered at least 3 h apart from other oral medications to reduce the possibility of drug–drug interactions. CKD chronic kidney disease, DM diabetes mellitus, eGFR estimated glomerular filtration rate, HF heart failure, K^+^ potassium, Mg magnesium, RAASi renin–angiotensin–aldosterone system inhibitor

The algorithm details serum K^+^ thresholds and patient profiles requiring treatment with novel potassium binders/patiromer (Fig. 1). Mild hyperkalemia (serum K^+^ 5.1–5.5 mEq/L) is considered acceptable due to low associated risk and should not limit RAASi titration. However, serum K^+^ levels between 5.1 – 5.5 mEq/L, with recurrent RAASi-associated hyperkalemia or in patients with HF and concomitant CKD stage 3b–4 and/or DM, are associated with increased mortality [5]. Similarly, serum K^+^ levels > 5.5 mEq/L (moderate and severe hyperkalemia) are associated with increased mortality risk. Hence, the algorithm advocates the use of novel potassium binders/patiromer to enable RAASi titration for both such circumstances. In patients who experience severe hyperkalemia (serum K^+^ > 6.0 mEq/L), RAASi therapy should be discontinued or reduced, and novel potassium binders/patiromer initiated. Following normalization of serum K^+^ levels, crucial RAASi therapy should be re-initiated and titrated to maximal doses with close monitoring of serum K^+^ levels.

Of note, every patient with a serum K^+^ level > 5.0 mEq/L should be referred to hospital for specialist advice [1]; novel potassium binders should not substitute emergency treatment for acute episodes of hyperkalemia and should only be initiated once the patient is in a stable condition.

Following titration of RAASi to maximal doses, close monitoring of serum K^+^ levels for hyperkalemia is recommended [1], the outcome of which may guide further treatment decisions. It is crucial to highlight that serial monitoring of serum K^+^ is endorsed by treatment guidelines [1] and followed in clinical trial protocols; however, this may not be reflected in clinical practice [2], increasing the risk of arrhythmias and sudden cardiac death due to hyperkalemia. Following initiation or up-titration of a novel potassium binder, serum K^+^ should be monitored at 48–72 h and repeated after 1 week, 1 month, and 3–6 months. Serum magnesium should also be monitored for at least 1 month following initiation of patiromer.

In addition to maximal RAASi dosing, the expert panel advocates interrogation of additional causes of hyperkalemia in all patients, including diet, potassium salt substitutes, and other medications that may impair renal function.

## Conclusions

The goal of this treatment algorithm is to provide further guidance to physicians on the treatment of RAASi-associated hyperkalemia in patients with HF and/or other CVD. The algorithm aims to enable maximal RAASi dosing, critical to achieve the greatest possible reduction in morbidity and mortality. Further evidence on the long-term benefits of patiromer in patients with HF receiving essential RAASi therapy is awaited from the DIAMOND trial.
